# Superconducting Coplanar Waveguide Filters for Submillimeter Wave On-Chip Filterbank Spectrometers

**DOI:** 10.1007/s10909-016-1579-8

**Published:** 2016-03-24

**Authors:** A. Endo, S. J. C. Yates, J. Bueno, D. J. Thoen, V. Murugesan, A. M. Baryshev, T. M. Klapwijk, P. P. van der Werf, J. J. A. Baselmans

**Affiliations:** Department of Microelectronics, Faculty of Electrical Engineering, Mathematics and Computer Science, Delft University of Technology, Mekelweg 4, 2628 CD Delft, The Netherlands; Kavli Institute of Nanoscience, Faculty of Applied Sciences, Delft University of Technology, Lorentzweg 1, 2628 CJ Delft, The Netherlands; SRON Netherlands Institute for Space Research, Landleven 12, 9747 AD Groningen, The Netherlands; SRON Netherlands Institute for Space Research, Sorbonnelaan 2, 3584 CA Utrecht, The Netherlands; Kapteyn Astronomical Institute, University of Groningen, P.O. Box 800, 9700 AV Groningen, The Netherlands; Physics Department, Moscow State Pedagogical University, 119991 Moscow, Russia; Leiden Observatory, Leiden University, PO Box 9513, 2300 RA Leiden, The Netherlands

**Keywords:** Spectroscopy, Filters, Submillimeter wave, Astronomical instrumentation, Microwave kinetic inductance detectors

## Abstract

We show the first experimental results which prove that superconducting NbTiN coplanar–waveguide resonators can achieve a loaded *Q* factor in excess of 800 in the 350 GHz band. These resonators can be used as narrow band pass filters for on-chip filter bank spectrometers for astronomy. Moreover, the low-loss coplanar waveguide technology provides an interesting alternative to microstrip lines for constructing large scale submillimeter wave electronics in general.

## Introduction

On-chip filterbank spectrometers that use superconducting resonators as narrow band pass filters are becoming more popular as the design for realizing next-generation low-resolution millimeter–submillimeter (mm-submm) wave (100–1000 GHz) spectrometers for astronomy [1–3]. The concept relies on the availability of superconducting microresonators with sufficiently high *Q* factors to achieve the required frequency resolution, and a transmission line with low enough losses to carry the signal from the antenna to the far end of the filterbank. In cases where transmission line resonators are used as the band pass filters, the two requirements are related; the internal (unloaded) $Q_\mathrm {i}$ of the resonator is associated to the transmission loss of the line through [4]

1 $$\begin{aligned} Q_i = \frac{\pi }{\alpha \lambda }, \end{aligned}$$

where $\alpha$ is the attenuation constant and $\lambda$ is the wavelength in the resonator. (Note that Eq. 1 holds only if $Q_\mathrm {i}$ is limited by the nominal transmission loss of the line, and not if losses at the ends of the resonator dominate.) For example, the DESHIMA spectrometer [1, 2] in development requires filters with a loaded $Q_\mathrm {l} = 500$, equal to the designed frequency resolution of $F/\Delta F= 500$, at 326–905 GHz. Because transmission lines that carry the signal from one element to the next are the most fundamental building blocks for high frequency electronics, there are many applications that would grossly benefit from a transmission line technology with low losses in the mm-submm band; among those are superconductor–insulator–superconductor (SIS) mixer devices [5], traveling wave kinetic inductance parametric amplifiers [6], and near-field microscopes [7].

Coplanar waveguides (CPWs) are one of the most widely used kinds of transmission lines for superconducting mm-submm electronics. The advantages of CPWs include: (1) it can be made with a metal film deposited directly on a crystalline dielectric substrate, thereby eliminating the presence of amorphous dielectric materials that can be lossy [8], (2) it is trivial to make a short to the ground, making it easy to realize $\lambda /4$ resonators. Another advantage of CPWs that is often quoted is the ease of fabrication because it is a ‘single layer’ structure, but this holds less for long lines that require airbridges [9, 10] to suppress the odd-mode excitation. Although the intended even-mode of the CPW is less radiative, the radiation loss per unit length increases rapidly as a function of frequency *F*; in the case of a perfect conductor with no losses and no kinetic inductance, the attenuation constant is approximately proportional [11] to $F^3$. This has been the main reason that previous attempts to develop an on-chip direct detection spectrometer have adopted microstrip lines and not CPWs [2, 3, 8] for their resonant filters, though microstrips have their own challenge to minimize material losses, especially in the higher-frequency submillimeter band [8].

In this paper, we revisit the use of CPWs as narrow band pass filters for on-chip filterbank spectrometers. In order to suppress radiation loss, we fabricate sub-micron lines using electron-beam lithography [12]. We also take advantage of the fact that the kinetic inductance of superconducting films suppresses radiation loss [13], because the fraction of energy carried as the kinetic energy of Cooper pairs is not radiative. We experimentally prove that it is possible to achieve a loaded $Q_\mathrm {l}$ in excess of 500 required for the 350 GHz band of DESHIMA, indicating that the intrinsic (unloaded) $Q_\mathrm {i}$ is even higher.

## Device Design and Fabrication

**Fig. 1:**
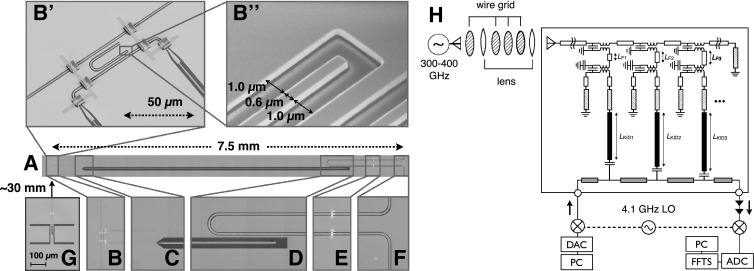
(*A*) Optical micrograph of one channel of the filterbank. Explanations of each section is given in the zoomed-in micrographs (*B*–*F*). (*B*) The intersection between the signal feed line, the filter, and the microwave kinetic inductance detector (MKID). The signal line carries the 300–400 GHz signal from the antenna (*G*). In between the signal line and the MKID to the right of the figure, there is the narrow band pass filter. (*B* $^\prime$) False-colored scanning electron micrograph of the same region as *B*, seen from an angle of 45$^\circ$. NbTiN, Al, polyimide, and the sapphire substrate are colored in *yellow*, *gray*, *green*, and *blue*, respectively. In the center of the image is a U-shaped $\lambda /4$ CPW resonator that acts as a narrow band pass filter. To the *bottom-right* of the filter is a small section of the MKID. The section that couples to the filter is made fully of NbTiN. After making a 90$^\circ$ turn on each side, the CPW center line is connected to a center line made of Al, where the submillimeter wave is absorbed. The 6 bridges visible in this image are placed to suppress the excitation of the odd mode due to asymmetry. Each bridge is made of Al on *top* of a block of polyimide. (*B* $^\prime \prime$) Further enlarged image of the open end of the filter. The *color coding* is the same as B$^\prime$, except that the unetched NbTiN remaining in the slot is colored with *red*. (*C*) On the *top* of the image is the shorted end of the MKID, where the Al center line is connected to the NbTiN ground plane. On the *bottom* of the image is the transition from the narrow CPW with Al center line and NbTiN ground plane, to the wide CPW made fully of NbTiN. (*D*) Coupler between the MKID and the microwave readout CPW, both made fully of NbTiN. (*E*) Polyimide-supported bridges of Al over the readout CPW. (*F*) Bend of the readout CPW, to the adjacent filters. (*G*) Optical micrograph of the double-slot antenna, with a different magnification than that of *B–F*. There is $\sim$30 mm of CPW length in between the antenna and the filter bank. (*H*) Block diagram representation of the filterbank chip (in the *large box*), and the experimental setup around it to measure the filter transmission as a function of frequency. *White rectangles* represent NbTiN CPWs with a center strip width of $S=0.6\ \mathrm {\upmu m}$ and a slot width of $W=1.0\ \mathrm {\upmu m}$. *Dashed rectangles* represent CPWs with a NbTiN ground plane and Al center strip, with $S = 1.4 \ \mathrm {\upmu m}$ and $W=2.3\ \mathrm {\upmu m}$. The *black rectangles* represent NbTiN CPWs with $S = 10 \ \mathrm {\upmu m}$ and $W=30\ \mathrm {\upmu m}$. The *gray rectangles* represent the readout CPW line made of NbTiN. Each channel of the filterbank has a different filter length $L_{\mathrm {F}n}$ and a different length $L_{\mathrm {KID}_n}$ of the wide NbTiN section of the MKID. For simplicity, only 3 out of the 13 channels are drawn in the figure and the rest are omitted. In addition to those, there were also 2 MKIDs with no connection to the signal line, used for subtracting the stray light contribution of the MKID response (Color figure online)

Micrographs of one channel of the filterbank are presented in Fig. 1A–G. An equivalent-circuit representation of the filterbank is included in Fig. 1H. Each channel is a combination of a filter, and a NbTiN/Al hybrid MKID [14]. The filter is a $\lambda /4$ resonator with one side open and the other side short circuited. The filter, as well as the $\sim$30 mm long signal line that carries the signal from the antenna to the filter bank, are made of a NbTiN CPW with a central line width of $S=0.6\,{\upmu }$m and a slot width of $W=1.0\,{\upmu }$m. The shorted end of the filter runs in parallel to the signal line, and the open end runs in parallel to the MKID. The filter transmission has been simulated using a commercial software Sonnet EM, to achieve a loaded $Q_\mathrm {i}$ of 560–615. After making a 90$^\circ$ turn on each side, the submm signal is guided to CPWs that have an Al center line to have the signal absorbed therein. The antenna is a double-slot antenna similar to the one adopted by Janssen et al. [14], backed with a Si lens with a diameter of 8 mm.

The device is fabricated on a 350 $\mathrm {\upmu m}$-thick *c*-plane sapphire substrate. After the wafer was cleaned in 85 vol% phosphoric acid at 110 $^\circ$C for 30 min, 350 nm of NbTiN was deposited by dc reactive sputtering of a NbTi target in an Ar and $\mathrm {N_2}$ plasma [15]. The pattern in the NbTiN, including the filter and signal line, was defined using electron-beam writing on PMMA resist, followed by an SF$_6$ + O$_2$ capacitively-coupled plasma etch and an O$_2$ plasma cleaning. The next step was the creation of the supporting blocks of the bridges, which was done by optical lithography of polyimide LTC9505 from Fujifilm. Finally, 50 nm of Al was sputter deposited, and patterned using contact-mask optical lithography and wet etching [9] to define the Al section of the MKIDs and also the bridges. Step coverage of the bridges is assured through the slightly sloped sides of the polyimide blocks (result of the negative-tone lithography), and the isotropy in the sputter deposition of Al.

## Measurement of the Filter *Q* Factor

The measurement of the filter *Q* was done in the same manner as that reported in our previous article [8], the only difference being the frequency of the narrow-band submillimeter wave source based on a $\sim$20 GHz synthesizer and frequency multipliers. The signal is attenuated by a series of wire grids, and goes through a window of a $\mathrm {^3He}$ sorption cryostat that cools the chip to 250 mK. While the submm signal frequency is swept from 300 to 400 GHz, the response of all MKIDs behind the filters, and 2 ‘blind’ MKIDs that are far away from the submm signal CPW, are simultaneously read out using an FFTS-based digital readout system [16]. The raw response of the MKIDs behind the filters has been divided with the response of one of the ‘blind’ MKIDs, to calibrate out the response to stray light that bypasses the filter bank and directly couples to the MKIDs. In the future, this stray light coupling needs to be eliminated by improving the design of the optical chain as well as the chip.

**Fig. 2:**
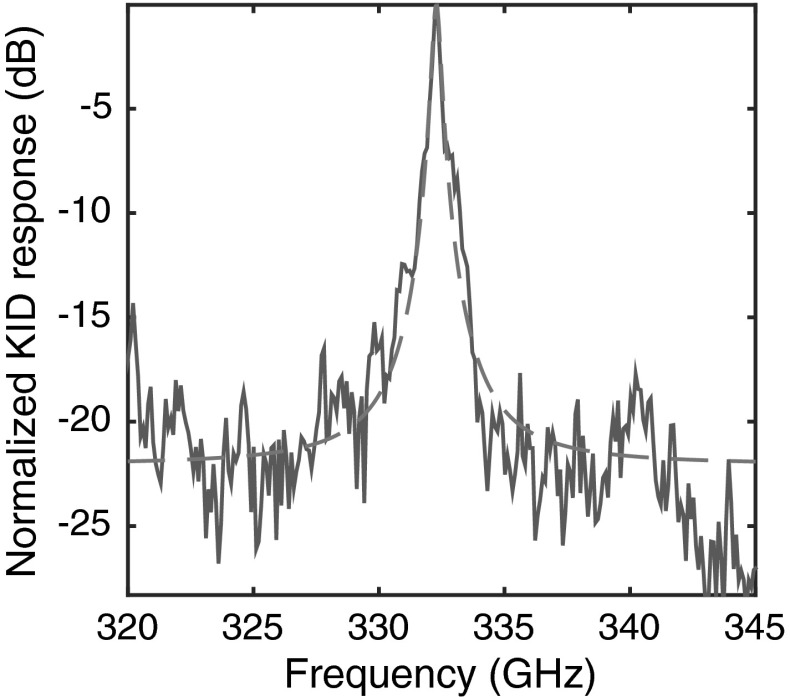
Normalized response of an MKID behind a filter. The *solid* (*blue*) *curve* is the data, where the *dashed* (*red*) *curve* is a Lorentzian fit to the data in the range of 315–415 GHz. From the fit, we deduce a loaded *Q* factor of $Q_\mathrm {l} = 849$ (Color figure online)

The response of one of the MKIDs behind a filter is presented in Fig. 2. From a Lorentzian fit to the transmission peak, we deduce a loaded *Q* factor of $Q_{\mathrm {l}}=849$. The median of the *Q* factor of all 9 channels that were measured was 516, which is close to the designed value of 560–615. The maximum and minimum $Q_{\mathrm {l}}$ values were 849 and 94, respectively. Because the median $Q_\mathrm {l}$ is close to the designed coupling $Q_\mathrm {c}$, we suspect that the loaded $Q_{\mathrm {l}}$ is limited by the coupling of the filter to the signal line and to the MKID, rather than by internal loss. The variation in the measured loaded $Q_{\mathrm {l}}$ could be attributed to the relatively large beam step size of 100 nm that was used for the electron-beam lithography. The fact that we see some residual NbTiN in the slots of the CPW as seen in Fig. 1 could also be playing a role. We are currently developing a fabrication process that uses a beam step size of 2 nm, and an inductively coupled plasma to etch the slots with higher anisotropy.

## Conclusion

We have developed $\lambda /4$ NbTiN CPW filters that achieve a loaded $Q_{\mathrm {l}}$ in excess of 800, which is higher than the frequency resolution of $F/\Delta F = 500$ that is targeted by some astronomical on-chip direct detection spectrometers in development. This opens up the possibility of making very wide band submillimeter wave on-chip filter bank spectrometers, up to $\times 3$ of the lowest frequency. According to Eq. 1, this *Q* factor gives an upper limit to the loss of a bare CPW of $\sim$3 dB per 10 cm at around 330 GHz, which makes CPWs an attractive alternative to microstrips at this frequency range. Further development of this technology could enable submillimeter wave filter bank spectrometers and other submillimeter wave electronic devices that operate up to 1.1 THz, the gap frequency of NbTiN.
